# Prevalence and Risk Factors of Hypertension Among Children Attending Out Patient Department of a Tertiary Care Hospital in Karachi

**DOI:** 10.7759/cureus.7957

**Published:** 2020-05-04

**Authors:** Muhammad Bilal, Abdul Haseeb, Alina Saeed, Aena Saeed, Palwasha Ghaffar

**Affiliations:** 1 Internal Medicine/Pediatrics, Dr. Ruth K. M. Pfau Civil Hospital, Karachi, PAK; 2 Internal Medicine, The Wright Center for Graduate Medical Education, Scranton, USA; 3 Internal Medicine, Ziauddin University, Karachi, PAK

**Keywords:** hypertension, children, opd, high blood pressure

## Abstract

Introduction: The childhood obesity epidemic has caused the global prevalence of hypertension (HTN) in children to increase from 2% to 4%. However, there is limited data regarding this issue in Pakistan. Hence this cross-sectional study aims to document the prevalence of HTN and its risk factors among children visiting the out patient department (OPD) of a government hospital in Karachi, which is one of the largest cities in Pakistan. Methods: One thousand children aged between 4 and 12 years who visited the OPD in October 2019 were included. Blood pressures (BPs) for each child were measured manually and recorded. Their guardians were then interviewed to assess the risk factors present in each child. Data collected were analyzed using SPSS (Statistical Package for the Social Sciences). Results: Among all the children, those between 4 and 7 years of age had a higher prevalence of HTN (19.2%; 9.2% stage 1 and 10.0% stage 2) than children aged between 8 and 12 years (14.5%; 8.0% stage 1 and 6.5% stage 2). Obese children between the age of 4 and 7 years (OR = 3.11) were more likely to develop HTN. Moreover, children with a positive family history of HTN were 1.43 times more likely to have HTN and 1.32 times more likely to have pre-HTN. There was no significant association of gender, artificial feeding, low birth weight, and maternal smoking with HTN. Conclusion: The prevalence is particularly higher in children aged between 4 and 7 years (19.2%) and there is a strong association between high BMI (body mass index), family history of HTN, and high-fat diet intake with HTN in children. There was no significant variation of prevalence between both genders.

## Introduction

Hypertension (HTN) in children has become a matter of concern and a rising issue in most parts of the world. For a long time, it had been underdiagnosed by the physicians because of the various ranges present which unlike adults vary with age, gender, and height [1]. However, this had led to the publication of the fourth report of NHLBI’s (National Heart, Lung, and Blood Institute) which states that a child is considered hypertensive when the blood pressure (BP) measured at three different occasions is at or above the 95th percentile of the normal curve. These percentiles are determined according to age, gender, and height of a child; each of which influences BP individually [2]. Moreover, due to the early cardiovascular changes including left ventricular hypertrophy and diastolic dysfunction, it is essential to diagnose a hypertensive child and try to prevent its long-term effects on the body [1]. Increased BP in childhood is a strong indicator of the development of HTN in adulthood. Multiple end-organ involvements like hypertensive retinopathy, microalbuminuria due to kidney damage, and learning disabilities are also some of the reported side effects of HTN during childhood [3]. The prevalence of high BP in adolescents is on a rise with it being between 1% and 5% currently in the United States, 16.4% in central Europe, around 30% in northern Greece, and 13.8% in China [4-7]. Moreover, in India, the hospital admission of children diagnosed with HTN doubled from 1997 to 2006 from 18 per 100,000 to 35 per 100,000 admissions respectively [8]. A study conducted in Karachi has shown the prevalence of HTN to be 3.0% and its positive correlation with high BMI (body mass index) among children [9]. Other than that limited data are available on the prevalence of HTN in adolescents in Pakistan. Several risk factors are associated with HTN in various studies, with obesity being a major one. The association between obesity and raised BP is further demonstrated by the fact that an overweight child is three times more likely to develop HTN than a child with a BMI in the normal range [10]. Along with obesity, high sodium intake, strong family history, increased fructose intake, and uric acid levels, male sex and ethnicity are some of the other established risk factors for the development of HTN in a child [10]. Therefore, the purpose of our study is to identify the risk factors and the prevalence of HTN among the pediatric age group attending the OPD (out patient department) of a tertiary care hospital in Karachi.

## Materials and methods

This study was conducted in the pediatric OPD of Dr. Ruth K. M. Pfau Civil Hospital Karachi, in October 2019 and included 1000 children who were randomly selected. All of them were aged between 4 and 12 years and attended the OPD for various complaints. They were screened for HTN by manually measuring their BPs at the hospital visit. The children along with their guardians or parents were also interviewed to evaluate the risk factors present in each of them. The questionnaire used was taken from a similar study that was conducted in a hospital in Saudi Arabia [11]. The patients who participated in the study were kept anonymous and no names were used in the survey or while forming the results. Written well-informed consent was taken from the guardian before the interview. The study and its protocols were approved by an ethical review board of Dow University of Health Sciences. The questionnaire consisted of three main sections; the first part included demographic details like age, gender and also possible risk factors for HTN such as nationality, family history of essential HTN, physical inactivity, high-fat diet intake, artificial feeding, low birth weight, obstructive sleep apnea (snoring during sleep), and maternal smoking. The second section evaluated the BMI of the child using their height and weight. According to the current guidelines a child is overweight if its BMI lies within the 85th-95th percentile of the normal values and is categorized as obese if its BMI > 95th percentile [12]. In the third section, the BPs were recorded by taking an average value of three readings for each child and then categorized according to the current recommendations. A child with normal BP lies under the 90th percentile, a prehypertensive child lies under the 95th percentile, and a child is defined hypertensive when the BP lies at or above the 95th percentile. This is further divided into stage 1 (>95th-<99th percentile) and stage 2 (>99th percentile) [13]. The data were evaluated and analyzed using SPSS (Statistical Package for the Social Sciences). The numerical data were assessed by calculating the mean and standard deviation for each variable. The qualitative data collected from the survey were categorized and frequencies or percentages were calculated to form the results. Different tests were then used on the data to form results and associations.

## Results

A total of 1000 children aged between 4 and 12 years participated in this study, out of which 700 (70.0%) were male and 300 (30.0%) were female. Table 1 shows the prevalence of HTN and pre-HTN among each gender and age group; in males, it was calculated to be 21.1% (10.0% stage 1 and 11.1% stage 2) and 12.0% respectively whereas in females it was lower with 19.3% (9.0% stage 1 and 10.3%) and 5.6% respectively. It also showed that children between the age of 4 and 7 years had a higher prevalence of HTN (19.2%; 9.2% stage 1 and 10.0% stage 2) than those between the age of 8 and 12 years (14.5%; 8.0% stage 1 and 6.5% stage 2).

**Table 1:** Demographic details and prevalence of HTN among study participants. HTN, hypertension

	HTN N (%)	Pre-HTN N (%)	Normal N (%)
	Stage 1 Stage 2		
Gender			
Male (N=700)	70 (10.0) 78 (11.1)	84 (12.0)	468 (66.9)
Female (N=300)	27 (9.0) 31(10.3)	17 (5.6)	225 (75)
Age (years)			
4-7 (N=600)	55 (9.2) 60 (10.0)	40 (6.7)	445 (74.2)
8-12 (N=400)	32 (8.0) 26 (6.5)	12 (3.0)	330 (82.5)

Table 2 shows the relationship between BP and risk factors leading to it. According to the results, there is a statistically significant difference between the BPs of different age groups (p=0.00), with there being a higher prevalence of HTN and pre-HTN of 25.0% and 10.0% respectively among children of 4-7 years of age. The data also show a significant association of BMI, family history of HTN, and high-fat diet intake with the prevalence of HTN. Among these, positive family history of HTN (p=0.00) has the most significant association with 20% of children having HTN and 10% of them having pre-HTN. However, some of the other demographics like gender, nationality, and low birth weight showed no significant difference in BPs (p > 0.05).

**Table 2:** Relationship between BP classification and study participants. HTN, hypertension; BP, blood pressure * indicates p value <0.05 and significant data

	HTN N (%)	Pre-HTN N (%)	Normal N (%)	p value
Gender				
Male (N=700)	140 (20.0)	7 (1.0)	553 (79.0)	0.890
Female (N=300)	60 (20.0)	12 (4.0)	228 (76.0)	
Age (years)				
4-7 (N=600)	150 (25.0)	60 (10.0)	390 (65.0)	0.001*
8-12 (N=400)	32 (8.0)	12 (3.0)	356 (89.0)	
Nationality				
Pakistani (N=700)	70 (10.0)	80 (11.4)	550 (78.5)	0.545
Non-Pakistani (N=300)	45 (15.0)	15 (5.0)	240 (80.0)	
BMI				
Overweight/Obese (N=200)	40 (20.0)	10 (5.0)	150 (75.0)	0.001*
Healthy body weight (N=800)	64 (8.0)	32 (4.0)	704 (88.0)	
Family history of HTN				
Yes (N=650)	130 (20.0)	65 (10.0)	455 (70.0)	0.001*
No (N=350)	35 (10.0)	17 (4.8)	298 (85.1)	
Limited physical activity				
Yes (N=200)	30 (15.0)	10 (5.0)	170 (85.0)	0.385
No ( N=800)	160 (20.0)	80 (10.0)	560 (70.0)	
High-fat diet intake				
Yes (N=550)	110 (20.0)	50 (9.1)	390 (70.9)	0.001*
No (N=450)	40 (8.9)	20 (4.4)	390 (86.7)	
Artificial feeding				
Yes (N=100)	15 (15.0)	10 (10.0)	75 (75.0)	0.989
No (N=900)	135 (15.0)	90 (10.0)	675 (75.0)	
Low birth weight				
Yes (N=150)	15 (10.0)	25 (16.7)	110 (73.3)	0.112
No (N=850)	90 (10.6)	50 (5.9)	710 (83.5)	
Maternal smoking				
Yes (N=50)	3 (6.0)	4 (8.0)	43 (86.0)	0.156
No ( N=950)	40 (4.2)	45 (4.7)	865 (91.1)	

Table 3 illustrates correlation of average systolic blood pressure (SBP) and diastolic blood pressure (DBP) with various characteristics of the sample population. The SBP shows a positive correlation with weight (R = 0.345, p = 0.00), height (R = 0.342, p = 0.00), and BMI (R =0.201, p = 0.04) but not with age (R = 0.078, p = 0.07). The DBP shows a statistically significant correlation with age (R = 0.132, p = 0.00), weight (R = 0.123, p = 0.00), and BMI (R = 0.232, p = 0.00) while showing no significant association with height (R = 0.089, p = 0.07).

**Table 3:** Correlation of average SBP and DBP with various characteristics of study participants. SBP, systolic blood pressure; DBP, diastolic blood pressure * indicates p value <0.05 and significant data

	SBP		DBP	
	R	p-value	R	p-value
Age	0.078	0.07	0.132	0.001*
Weight	0.345	0.001*	0.123	0.001*
Height	0.342	0.001*	0.089	0.07
BMI	0.201	0.04*	0.232	0.001*

Table 4 shows the correlation of each risk factor with HTN and pre-HTN. Obese children (adjusted odds ratio, AOR = 3.11 at p = 0.03) between the age of 4 and 7 years (AOR = 3.11 at p = 0.00) were most likely to develop HTN according to the results shown in Table 4 as compared to the children with normal BMI who are aged between 8 and 12 years. Children with a positive family history of HTN were 1.43 times more likely to develop HTN (0.04) and 1.32 times more likely to have pre-HTN (p = 0.02). Those with low birth weight and limited physical activity had a higher chance of developing HTN with an AOR of 1.54 and 0.54 respectively. Moreover, children between the age of 4 and 7 years were likely to have pre-HTN (p = 0.04) than any of the other groups with an AOR of 4.21. Male gender, high-fat diet intake, artificial feeding, and low birth weight were some of the common predictors of developing pre-HTN among the children with each having an AOR of 1.30, 1.32, 1.67, and 1.32 respectively.

**Table 4:** Multiple logistic regressions demonstrating risk factors for HTN and pre-HTN. HTN, hypertension; AOR, adjusted odds ratio; CI, confidence interval; BMI, body mass index * indicates p value <0.05 and significant data

	HTN				Pre-HTN			
	AOR	p-value	95% CI		AOR	p-value	95% CI	
			Lower	Upper			Lower	Upper
Gender								
Male	0.91	0.32	0.31	1.88	1.30	0.88	0.44	2.98
Age								
(4-7)	3.11	0.001*	1.11	6.23	4.21	0.04*	1.76	5.67
BMI								
Obese	3.11	0.03*	1.11	2.31	0.87	0.91	0.12	3.89
Family history of HTN								
Yes	1.43	0.04*	1.88	3.31	1.32	0.02*	1.32	4.43
Limited physical activity								
Yes	0.54	0.89	0.32	1.67	0.55	0.87	0.12	3.23
High-fat diet intake								
Yes	0.65	0.45	0.43	2.32	1.32	0.54	0.21	3.23
Artificial feeding								
Yes	0.11	0.54	0.65	3.21	1.67	0.54	0.44	2.12
Low birth weight								
Yes	1.54	0.23	0.88	1.99	1.32	0.66	0.77	3.56

## Discussion

The most significant finding in our study is the higher prevalence of HTN and pre-HTN in children aged between 4 and 7 years which was found to be 25.0% and 10.0% respectively. This can be linked to the genetic predisposition of children for developing HTN or can also be explained by the rise in BMI that occurs at this age after an initial decline in weight starting from the first year of life [14]. This phenomenon is termed as ‘Adiposity rebound’ and is a strong indicator of developing obesity and its complications later in life [15]. The increase in BMI is linked to HTN and hence the adiposity rebound in children of this age group explains the high prevalence of elevated BPs found in our study [16]. Similar results are also seen in a study conducted in the United States in this age range [17]. Our finding is also consistent with a study conducted in European countries showing a high prevalence of about 20.0% in children aged between 2 and 9 years [18]. Another study conducted in Canada showed a very high prevalence of HTN (24.8%) in children aged 3-8 years [17]. On the contrary, some studies have shown an increase in the prevalence of HTN with age [19-21]. Such results may have been reported as these studies had a lower prevalence of obesity in children as compared to ours. It is also important to evaluate the risk factors leading to the development of elevated BPs in the young age group as BP consistently higher than the 90th percentile leads to signs of end-organ damage in early adulthood [22]. We evaluated certain risk factors in our study including age, gender, BMI, high-fat diet intake, family history of HTN, artificial feeding, low birth weight, etc. The results of our study showed another strong association of HTN with high-fat diet intake in children (20.0% each). This finding is similar to those found in studies conducted in Nigeria, India, and Greece [23-26]. Obesity in children due to decreased physical activity, high-fat dietary intake, and a sedentary lifestyle can explain this increased prevalence of elevated BPs in this group [18]. A family history of HTN also proved to be a strong indicator in our study where 20.0% of children having a positive family history had HTN and 10.0% had pre-HTN. This finding is consistent with an Indian and a Nigerian study [27-28]. There has been no gender or nationality variation in our study. There is some limitation to our study. Firstly, the BP was measured at a single occasion and this may lead to an overestimation of results as it includes a white coat and masked HTN. Secondly, this study was conducted in a government hospital of one city which may not be a complete representation for the entire country. Lastly, as our study is cross-sectional hence temporal association between the risk factors and HTN cannot be established.

## Conclusions

Our study shows that prevalence is particularly higher in children aged between 4 and 7 years and there is a strong association between high BMI, family history of HTN, and high-fat diet intake with HTN in children. There was no significant variation of prevalence between both genders. This analysis further suggests that large-scale screening of young children is required to report the prevalence of elevated BPs among them. Hence, dealing with the modifiable risk factors among them can contribute to lowering the development of chronic debilitating illnesses among adults resulting from long-standing HTN.

## References

[1] Hansen ML, Gunn PW, Kaelber DC (2007). Underdiagnosis of hypertension in children and adolescents. JAMA.

[2] U.S. Department of Health and Human Services, National Institutes of Health, National Heart, Lung Lung, and Blood Institute (2005). The Fourth Report on the Diagnosis, Evaluation, and Treatment of High Blood Pressure in Children and Adolescents. https://www.nhlbi.nih.gov/files/docs/resources/heart/hbp_ped.pdf.

[3] Bucher BS, Ferrarini A, Weber N (2013). Primary hypertension in childhood. Curr Hypertens Rep.

[4] Ahern D, Dixon E (2015). Pediatric hypertension: a growing problem. Prim care.

[5] Martin L, Oepen J, Reinehr T (2015). Ethnicity and cardiovascular risk factors: evaluation of 40,921 normal-weight, overweight or obese children and adolescents living in Central Europe. Int J Obes (Lond).

[6] Mavrakanas T, Konsoula G, Patsonis I (2009). Childhood obesity and elevated blood pressure in a rural population of northern Greece. Rural and Remote Health.

[7] Xi B, Liang Y, Mi J (2013). Hypertension trends in Chinese children in the national surveys, 1993 to 2009. Int J Cardiol.

[8] Amritanshu K, Kumar A, Pathak A (2015). Prevalence and risk factors associated with hypertension in children and adolescents. Pediatr Oncall J.

[9] Rahman AJ, Qamar FN, Ashraf S (2013). Prevalence of hypertension in healthy school children in Pakistan and its relationship with body mass index, proteinuria and hematuria. Saudi J Kidney Dis Transpl.

[10] Orlando A, Cazzaniga E, Giussani M (2018). Hypertension in children: role of obesity, simple carbohydrates, and uric acid. Front Public Health.

[11] Ghamri RA, Hegazy AA, Azizkhan AZ (2019). High blood pressure in children attending pediatric clinic at King Abdulaziz University Hospital, Jeddah, Saudi Arabia. J Fam Commun Med.

[12] (2000). Centers for Disease Control National Center for Health Statistics. Body mass index (BMI) percentiles for boys, 2 to 20 years.

[13] Riley M, Bluhm B (2012). High blood pressure in children and adolescents. Am Fam Phys.

[14] Allison DB, Heshka S, Heymsfield SB (1993). Evidence of a major gene with pleiotropic action for a cardiovascular disease risk syndrome in children younger than 14 years. Am J Dis Child.

[15] Rolland-Cachera MF, Deheeger M, Bellisle F (1984). Adiposity rebound in children: a simple indicator for predicting obesity. Am J Clin Nutr.

[16] Kannel WB, Brand N, Skinner JJ Jr (1967). The relation of adiposity to blood pressure and development of hypertension. The Framingham study. Ann Intern Med.

[17] Eisenmann JC, Wrede J, Heelan KA (2005). Associations between adiposity, family history of CHD and blood pressure in 3-8 year-old children. J Hum Hypertens.

[18] de Moraes AC, Carvalho HB, Siani A (2015). Incidence of high blood pressure in children - effects of physical activity and sedentary behaviors: the IDEFICS study: high blood pressure, lifestyle and children. Int J Cardiol.

[19] Amadi OF, Okeke IB, Ndu IK (2019). Hypertension in children: could the prevalence be on the increase?. Niger Med J.

[20] Basiratnia M, Derakhshan D, Ajdari S (2013). Prevalence of childhood obesity and hypertension in south of Iran. Iran J Kidney Dis.

[21] Dobson CP, Eide M, Nylund CM (2015). Hypertension prevalence, cardiac complications, and antihypertensive medication use in children. J Pediatr.

[22] Iang X, Srinivasan SR, Radhakrishnamurthy B (1994). Microalbuminuria in young adults related to blood pressure in a biracial (black-white) population. The Bogalusa Heart Study. Am J Hypertens.

[23] Noubiap JJ, Essouma M, Bigna JJ (2017). Prevalence of elevated blood pressure in children and adolescents in Africa: a systematic review and meta-analysis. Lancet Public Health.

[24] Uwaezuoke SN, Okoli CV, Ubesie AC (2016). Primary hypertension among a population of Nigerian secondary school adolescents: Prevalence and correlation with anthropometric indices: a cross-sectional study. Niger J Clin Pract.

[25] Sundar JS, Adaikalam JMS, Parameswari S (2013). Prevalence and determinants of hypertension among urban school children in the age group of 13-17 years in Chennai, Tamilnadu. Epidemiology.

[26] Karatzi K, Protogerou AD, Moschonis G (2017). Prevalence of hypertension and hypertension phenotypes by age and gender among schoolchildren in Greece: the healthy growth study. Atherosclerosis.

[27] Mijinyawa MS, Iliyasu Z, Borodo MM (2008). Prevalence of hypertension among teenage students in Kano, Nigeria. Niger J Med.

[28] Patel A, Bharani A, Sharma M (2019). Prevalence of hypertension and prehypertension in schoolchildren from Central India. Ann Pediatr Cardiol.

